# The joint protective function of live- and dead-*Lactobacillus plantarum* GKD7 on anterior cruciate ligament transection induces osteoarthritis

**DOI:** 10.18632/aging.206101

**Published:** 2024-09-05

**Authors:** Yen-You Lin, Chih-Ying, Wu, You-Shan Tsai, Chin-Chu Chen, Tzu-Ching Chang, Li-Chai Chen, Hsien-Te Chen, Chin-Jung Hsu, Chih-Hsin Tang

**Affiliations:** 1 Department of Pharmacology, School of Medicine, China Medical University, Taichung, Taiwan; 2 Department of Neurosurgery, China Medical University Hospital, Taichung, Taiwan; 3 Department of Neurosurgery, China Medical University Hsinchu Hospital, Hsinchu, Taiwan; 4 Biotech Research Institute, Grape King Bio Ltd., Taoyuan, Taiwan; 5 Institute of Food Science and Technology, National Taiwan University, Taipei, Taiwan; 6 Department of Pharmacy, Tajen University, Pingtung, Taiwan; 7 Department of Sports Medicine, College of Health Care, China Medical University, Taichung, Taiwan; 8 Department of Orthopedic Surgery, China Medical University Hospital, Taichung, Taiwan; 9 School of Chinese Medicine, China Medical University, Taichung, Taiwan; 10 Chinese Medicine Research Center, China Medical University, Taichung, Taiwan; 11 Department of Medical Laboratory Science and Biotechnology, College of Medical and Health Science, Asia University, Taichung, Taiwan; 12 Department of Medical Research, China Medical University Hsinchu Hospital, Hsinchu, Taiwan

**Keywords:** osteoarthritis (OA), *Lactobacillus plantarum* GKD7, metalloproteinase (MMP)3, collage II, aggrecan

## Abstract

Osteoarthritis (OA) is a chronic inflammatory disease accompanied by joint pain, bone degradation, and synovial inflammation. Tumor necrosis factor (TNF)-α and interleukin (IL)-1β play key roles in chronic inflammation, and matrix metalloproteinase (MMP)3 is the first enzyme released by chondrocytes and synovial cells that promotes MMPs’ degrading cartilage matrix (including collage II and aggrecan) function. Using an anterior cruciate ligament transection (ACLT) rat model, *Lactobacillus plantarum* GKD7 has shown anti-inflammatory and analgesic properties. The present investigation examined the chondroprotective effects of several dosages and formulas of GKD7 on rats in an ACLT-induced OA model. The findings indicate that oral treatment with both live-GKD7 (GKD7-L) and dead-GKD7 (GKD7-D), along with celecoxib (positive control), all reduce post-ACLT pain and inflammation in OA joints. Subsequently, the immunohistochemical staining results demonstrate that following GKD7-L and GKD7-D treatment, there was a reversal of the degradation of collagen II and aggrecan, as well as a decrease in the expression of IL-1β and TNF-α on the synovial tissue and MMP3 on the cartilage. Accordingly, our findings imply that the treatment of both GKD7-L and GKD7-D has chondroprotective and analgesic effects on the OA rat model, and that celecoxib and GKD7-L at dosages (100 mg/kg) have comparable therapeutic benefits. As a result, we propose that both GKD7-L and GKD7-D are helpful supplements for OA management.

## INTRODUCTION

Osteoarthritis (OA) is a degenerative total joint disease that develops with age or following trauma, and OA is a significant contributor to disability and the most prevalent illness that impairs function [1, 2]. Due to the combined effects of global population aging and rising obesity rates, OA is on the rise and is currently predicted to impact 303 million people globally [3, 4]. As the primary symptoms of OA, cartilage degradation is associated with bone lesions, synovial inflammation, and chronic pain [5, 6]. Normal, quiescent chondrocytes become activated and undergo a phenotypic shift during the development of OA. This leads to increased cartilage calcification associated with tidemark advancement or duplication, chondrocyte clusters forming, fibrillation and degradation of cartilage matrix, and vascular penetration from the subchondral bone [7]. The production of matrix breakdown products by concurrent overexpression of cartilage-degrading proteinases can further encourage catabolic activation, abnormal, hypertrophy-like differentiation, and death [8]. The collagen network cannot be restored to its pre-damaged state once it has occurred [9]. Preventing damage or promoting repair to restore the physiological and functional characteristics of the original cartilage is therefore the therapeutic challenge.

Chondrocytes are the only cells present in cartilage, which can be affected by pro-inflammatory cytokines, such as interleukin (IL)-1β and tumor necrosis factor (TNF)-α, which trigger the development of aggrecanases, MMPs, and other catabolic proteins [10–13]. When chemically and biomechanically damaging stimuli accumulate, OA chondrocytes react by going into a hypertrophic-like state that is marked by an increase in the activity of degradative enzymes [14] to break down a variety of cartilage extracellular matrix components (such as collagen II and aggrecan). And MMP3 is one of the MMPs which can stimulate the pro-forms of many other MMPs and aid in the activation of aggrecanase II in cartilage [15, 16]. Several studies have demonstrated that MMP3 levels are elevated in a variety of joint pathologies, including OA and acute injury [17–20]. On the other hand, MMP3 seems to be a significant and helpful therapeutic target in addition to IL-1β and TNFα for the development of therapeutic drugs for preventing OA cartilage deterioration.

Since probiotics are considered safe for consumption and many benefit from bioactivity for human disease, probiotics was the popular research and development objects for OA treatment. Probiotic strains of *Lactobacillus plantarum* is a well-studied probiotic bacterium that have been shown to reduce inflammation in human hosts, among their many other positive effects [21]. Thus, *Lactobacillus plantarum* have been applied to study on different human disease on clinical and pre-clinical trials, such as inflammatory bowel disease, abdominal pain, arthritis, and osteoporosis [22–24]. In our previous study we have already demonstrated that oral GKD7-L improved the progress of OA with reducing IL-1β and TNF-α production of OA [25]. In present study, we further investigate the chondrocyte protective effects of GKD7 with different dosages and formulas, including live and dead type for in future wide application on OA.

## MATERIALS AND METHODS

### Materials

IL-1β antibody (MAB601; final dilution of 1:200) was bought from R&D Systems, Inc. (Minneapolis, MN, USA). TNF-α (A11534; final dilution of 1:200) and collagen II (A1560; final dilution of 1:200) antibody were bought from ABclonal, Inc. (Woburn, MA, USA). MMP3 antibody (SC-21732; final dilution of 1:100) was bought from Santa Cruz Biotechnology (Dallas, TX, USA). Aggrecan antibody (ab3778; final dilution of 1:100) was bought from Santa Cruz Biotechnology (Dallas, TX, USA). All additional substances that were not previously listed were acquired from Sigma-Aldrich (St. Louis, MO, USA).

### Preparation of *Lactobacillus plantarum* GKD7

Live*-Lactobacillus plantarum* GKD7 (GKD7-L) isolated from Taiwanese pickles was cultured in de Man-Rogosa-Sharpe (MRS) medium for *lactobacilli* (Merck, Darmstadt, Germany) at 37°C for 16 h. To prepare GKD7-L freeze-dried powder, 0.03% of seed culture was scaled-up in a 15-ton bioreactor with a 12-ton working volume in a culture medium containing 5% glucose, 2% yeast extract, 0.05% MgSO_4_, 0.1% K_2_HPO_4_, and 0.1% Tween 80. After 16 h of incubation at 37°C, pellets of fermented bacteria were harvested by centrifugation, washed twice with reverse osmosis (RO) water then lyophilized with skim milk. The freeze-dried *L. plantarum* GKD7 powder contained approximately 5 × 10^11^ CFU/g live bacteria, according to the plate count method. To obtain the dead-*L. plantarum* GKD7 freeze-dried powder (GKD7-D), the GKD7-L pellets were heated under 121°C for 15 min then lyophilized. The resulting GKD7 powder, intended for use in the subsequent study, was administered at dosages of 100 mg/kg for both GKD7-L and GKD7-D, and 25 mg/kg for GKD7-L.

### OA rat model

Eight-week-old Sprague Dawley (SD) rats (300–350 g) were supplied by LASCo Inc. (Taipei, Taiwan) and maintained in an animal center under the Institutional Animal Care and Use Committee (IACUC) Guidelines issued by China Medical University (approval number: CMUIACUC-2023-283). Anterior cruciate ligament transection (ACLT) or sham surgery followed the procedures described in previous studies [26–28]. And rats were randomly divided into six groups: (1) sham operation without treatment (Control); (2) ACLT without treatment (OA); (3) OA treated with GKD7-L (25 mg/kg), GKD7-L (100 mg/kg), GKD7-D (100 mg/kg), and OA treated with celecoxib (50 mg/kg). GKD7 and celecoxib were administered orally five days per week. GKD7 and celecoxib, specifically, were taken orally five days a week. In all four research groups, the rats received 1 milliliter (ml) of reverse osmosis (RO.) water as a supplement beginning two days following surgery. In the ACLT-treated groups GKD7-L (at levels of 25 and 100 mg/kg) and GKD7-D were added to the RO. water for oral administration for six weeks. Furthermore, during the same six-week period, celecoxib (at a dose of 50 mg/kg) was dissolved in the RO. water and administered orally, except for the ACLT group that did not receive treatment. After 6 weeks, the rats were sacrificed.

### Weight-bearing testing

Postural deficiencies and spontaneous discomfort were assessed every week using the static weight-bearing Incapacitance Test (Bioseb, Paris, France). The rats were kept in an inclined plastic box with their hind paws placed on different sensors to measure the dynamic weight bearing between the limbs for a 10-second period. The grams were used to express the results. Our previously developed procedures [25, 27] were used. The following formula was used to determine the force value: (Force = weight on left limb – weight on right limb).

### Micro-CT analysis

Following CO_2_ sacrifice, the rats’ right knee joints were removed and preserved with 4% paraformaldehyde for micro-CT imaging and analysis [29, 30]. Using the settings chosen in previous studies, samples were photographed using a high-resolution micro-CT scanner (Skyscan 2211; Bruker, Kontich, Belgium) [27, 31], and InstaRecon^®^ software (Version v.1.3.9.2, Bruker micro-CT, Kontich, Belgium) was used for image reconstruction. After reorienting the reconstruction cross-sections and manually selecting 59 slices (0.5 mm), areas of interest (ROI) were created in accordance with our previous studies [27, 31]. The CTAn software was utilized to analyze several bone microarchitectural parameters, such as bone mineral density (BMD), bone mineral content (BMC), bone mineral thickness (Tb.Th), bone mineral separation (Tb.Sp), and bone mineral number (Tb.N) (Version 1.18.4, Bruker micro-CT, Kontich, Belgium) following our previous studies [27, 31].

### Histopathological analysis

The specimens undergo a 14-day decalcification process using 10% EDTA in PBS, a progressive ethanol concentration dehydration, and paraffin embedding for histological examination. Section slides were subsequently produced along the sagittal plane using Hematoxylin and Eosin (H&E) or Safranin-O/Fast-green, in accordance with previously published techniques [25, 27, 32]. According to our previous research, we looked at the Osteoarthritis Research Society International (OARSI) score, cartilage score, and synovium inflammation [25, 27]. The medial tibial plateau (the weight-bearing area) was the subject of an evaluation using the OARSI histopathology grading system. The system defined the grade of damage as the depth of osteoarthritis progression into the cartilage, ranging from 0 to 6, and the stage of damage as the horizontal extent of cartilage damage, ranging from 0 to 4. As in earlier research, the final score (grade × stage) ranges from 0 (normal cartilage) to 24 points (highest advanced grade and most extensive stage). As previously reported in our research [25, 27], cartilage deterioration was classified from “none” to “severe” (numerical values 0 to 5). Similarly, surgery-induced inflammation of the synovial membrane was graded from 0 to 4 [25, 27].

### Immunohistochemistry (IHC) staining

IHC staining data were quantified using methods documented in our previous method [12, 33, 34]. Briefly, the tissues were treated with primary antibodies. Secondary antibody binding and DAB staining were conducted using a Leica Novolink Polymer Detection system (Leica Biosystems Inc., Deer Park, IL, USA) [35, 36]. The final staining scores were analyzed by summing the intensity and percentage scores [25, 27]. Briefly, we used a microscope to gather photographs, and then we scored each image’s intensity as follows: 1 denotes low intensity, 2 denotes moderate intensity, and 3 denotes high intensity staining. We also determined the proportion of positive cells: positive cells range from 0% in case of 0 to <10% in case of 1, 10–29% in case of 2, 30–59% in case of 3, and 60–100% in case of 4. Intensity and percentage were combined to get a final score that ranged from 0 to 7 [25, 27].

### Statistical analysis

All information is presented as the mean ± SD, and GraphPad Prism 8 (Dotmatics, Boston, MA, USA) software was used for analysis. The unpaired two-tailed Student’s *t*-test, one-way analysis of variance, and Student-Newman-Keuls post hoc testing were used to assess the significant difference between the three groups. When a *p*-value was less than 0.05, it was deemed statistically significant.

## RESULTS

### Oral administration GKD7-L and GKD7-D improve joint pain without affecting body weight

**Figure 1:**
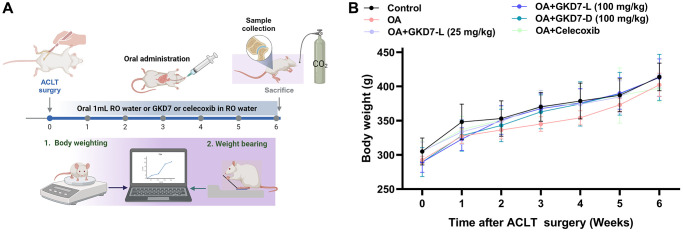
**Oral administration of GKD7-L and GKD7-D without affecting body weight in the OA rat model.** (**A**) The experimental design. (**B**) The GKD7-L or GKD7-D oral delivery did not significantly alter the body weights of OA rats as compared to controls.

**Figure 2:**
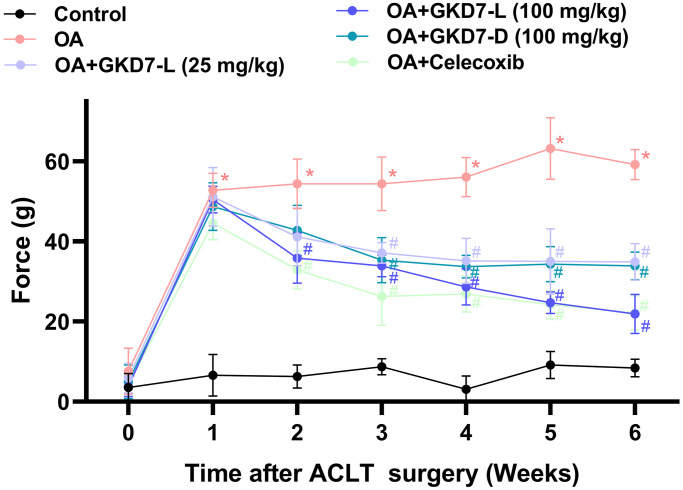
**Oral administration of GKD7-L and GKD7-D improved weight-bearing deficits after ACLT surgery.** Compared to OA-only rats, oral administration of GKD7-L and GKD7-D contributed to a considerable reduction in weight-bearing asymmetry following ACLT surgery. ^*^p < 0.05 versus controls; ^#^*p* < 0.05 versus the OA group.

ACLT-induced rat model is a popular approach for assessing anti-osteoarthritic activity in medicine or health food development [37]. In present study, we evaluate the chondroprotective activity of GKD7 on this rat model with oral administration GKD7-L, GKD7-D and celecoxib (Figure 1A). Throughout the trial, the body weights of each group climbed gradually, and no discernible changes were seen between them (Figure 1B), indicating that there were no negative side effects associated with GKD7-L and GKD7-D. Additionally, the weight-bearing test demonstrates that the ACLT rats’ weight-bearing posture showed a discernible asymmetry that worsened throughout the course of the trial’s first week (Figure 2). Furthermore, using different dosages and formulas of GKD7 improve with joint pain in the weight-bearing incapacitance test (Figure 2). This finding implies that both GKD7-L and GKD7-D can lessen OA-related joint pain, and that celecoxib’s anti-analgesic effects are comparable when taken in dosage (100 mg/kg) of GKD7-L.

### GKD7-L and GKD7-D both improve ACLT-induced bone erosion

**Figure 3:**
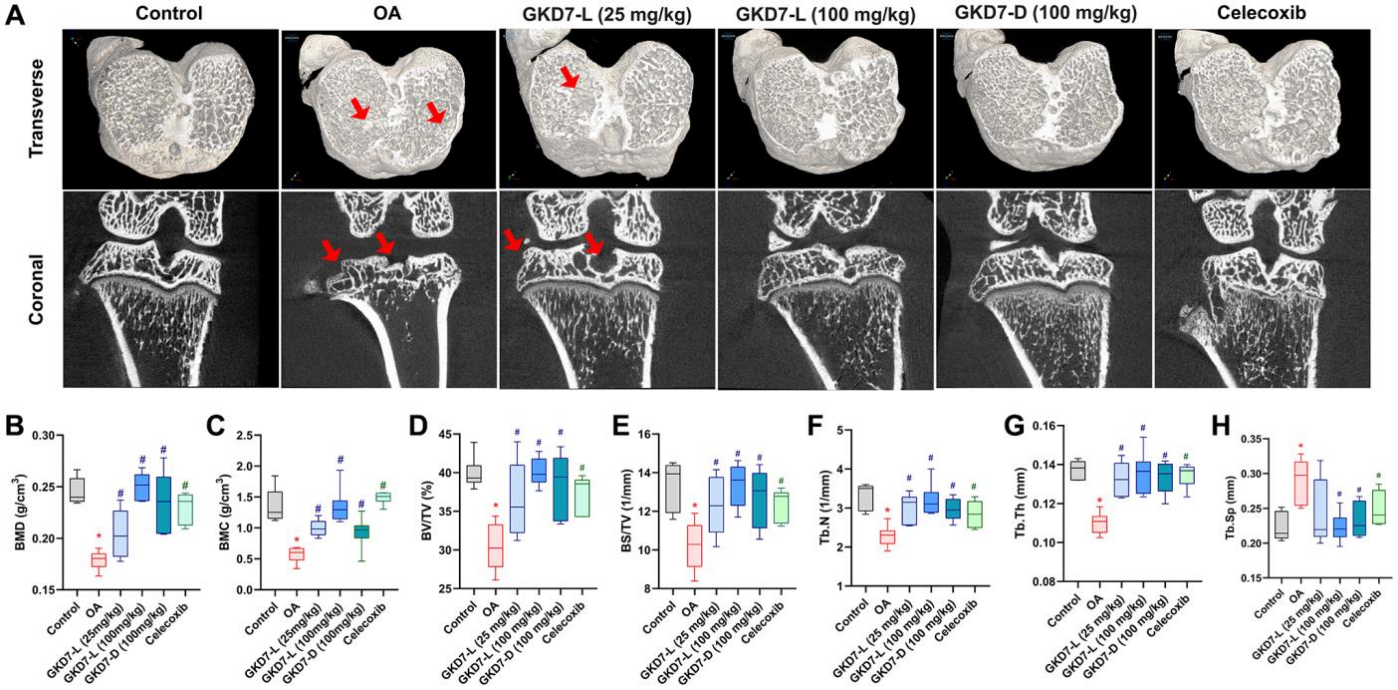
**Therapeutic effects of GKD7 treatment evaluated by micro-CT.** (**A**) Rat knee coronal and transverse micro-CT investigates from the control, OA, OA +GKD7-L (25 mg/kg), OA +GKD7-L (100 mg/kg), and OA +GKD7-D (100 mg/kg) groups. (**B**–**H**) Quantitative evaluations of bone surface/total volume (BS/TV), bone volume/total volume (BV/TV), bone mineral density (BMD), bone mineral content (BMC), bone volume/total volume (BV/TV), trabecular thickness (Tb.Th), trabecular number (Tb.N), and trabecular separation (Tb.Sp). The red arrows indicate bone erosion. ^*^p < 0.05 in comparison to the controls; ^#^*p* < 0.05 in comparison to the OA group.

In clinical and pre-clinical trial, the micro-CT evaluation is a powerful tool for diagnosing OA [38, 39]. As a result, we collected a rat knee sample for micro-CT imaging and analysis. The micro-CT images of the OA group clearly display bone loss (Figure 3A). Following ACLT-induced bone resorption, oral treatment of GKD7-L (25 and 100 mg/kg), GKD7-D (100 mg/kg), and celecoxib all show a reduction in obvious bone erosion with a significant difference on parameter analysis of micro-CT images (Figure 3B–3H). As a result, GKD7-L and GKD7-D administration at various doses and formulations stops bone degradation on ACLT-induced OA.

### Both GKD7-L and GKD7-D improve the histopathological characteristic of OA

**Figure 4:**
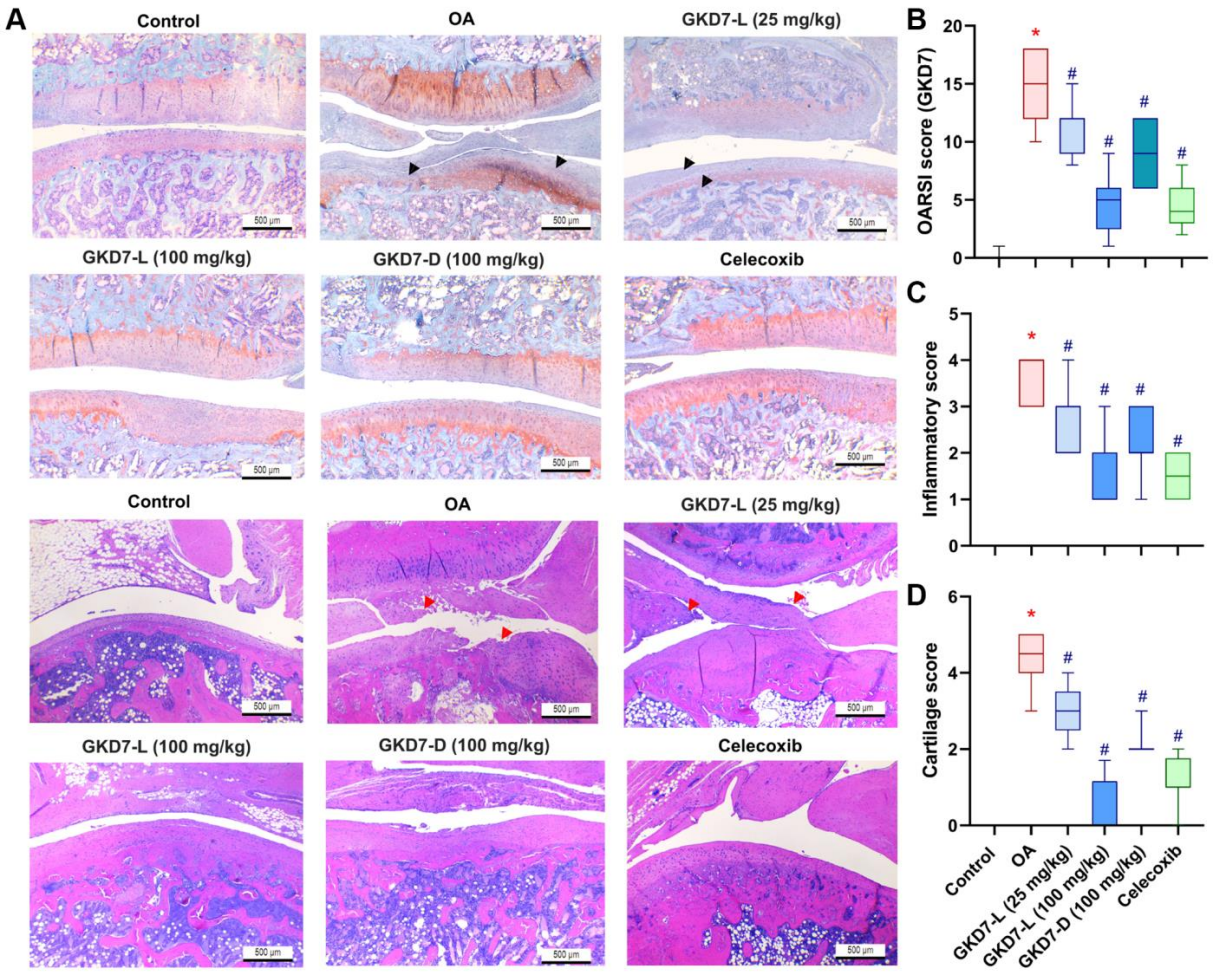
**Therapeutic effects of GKD7 treatment evaluated by histopathology.** (**A**) H&E and Safranin-O/Fast Green staining coronal images of articular cartilage from representative knee joints in each group (magnification 4x). Quantitative analyses of (**B**) Osteoarthritis Research Society International (OARSI) scores, (**C**) cartilage scores, and (**D**) synovium scores. The black arrows indicate the cartilage destruction. The red arrows indicate the synovial inflammation. Scale bar = 500 µm. ^*^p < 0.05 versus controls; ^#^*p* < 0.05 versus the OA group.

For more further investigation of the anti-osteoarthritic activity of GKD7, histopathological assessments were used for this study. The cartilage damage and synovial inflammation are the main histopathological characteristic of OA [40]. Furthermore, the cartilage degradation and synovial inflammatory response on the ACLT-induced animals are depicted in our histological images (Figure 4A). Additionally, there is a substantial difference between the OARSI score, cartilage score, and inflammatory score when compared to the control group. Administration of GKD7-L (25 and 100 mg/kg), GKD7-D (100 mg/kg), and celecoxib treatment all reversed the ACLT-induced the increasing of OARSI score, cartilage score, and inflammatory score (Figure 4B–4D). Therefore, the histological characteristics of OA can be improved by oral administration of GKD7-L and GKD7-D at different dosages and formulas.

### GKD7 improve joint matrix degradation via inhibiting MMP3, IL-1β and TNF-α

**Figure 5:**
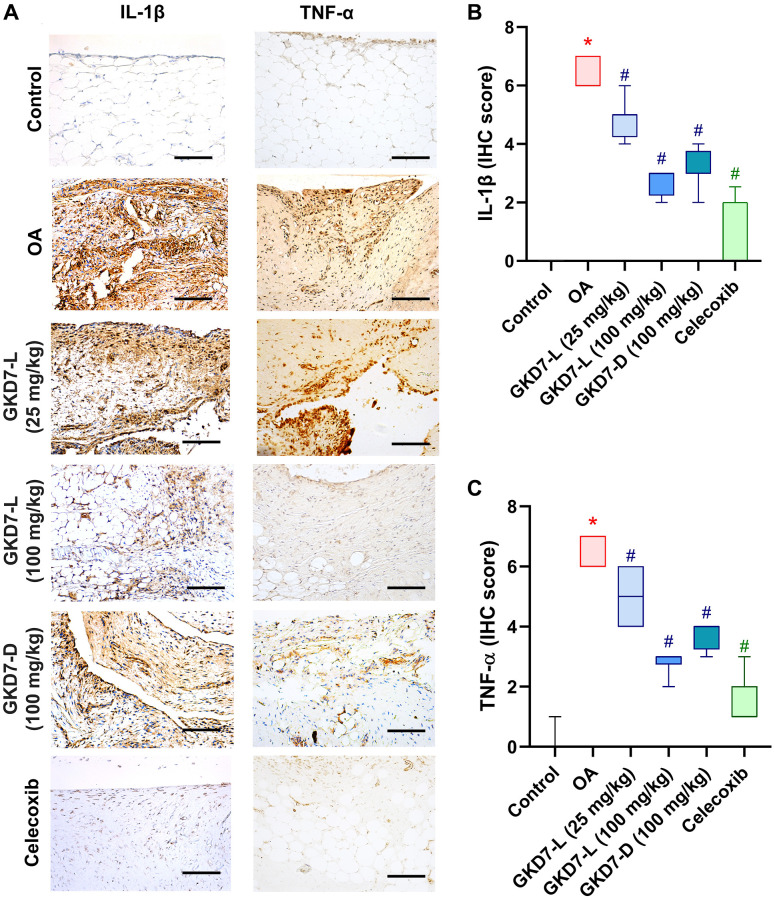
**Administration of GKD7-L and GKD7-D reduced levels of TNF-α and IL-1β expression on synovial tissue.** (**A**) IHC staining of TNF-α and IL-1β expression in a representative synovial tissue sample from the control, OA, OA+GKD7-L (25 mg/kg), OA+GKD7-L (100 mg/kg), and OA+GKD7-D (100 mg/kg) groups. Quantitative evaluations of cartilage’s TNF-α and IL-1β (**B**, **C**). Values are presented as the mean ± SD for each group. Scale bar = 100 µm. ^*^p < 0.05 versus controls; ^#^*p* < 0.05 versus the OA group.

**Figure 6:**
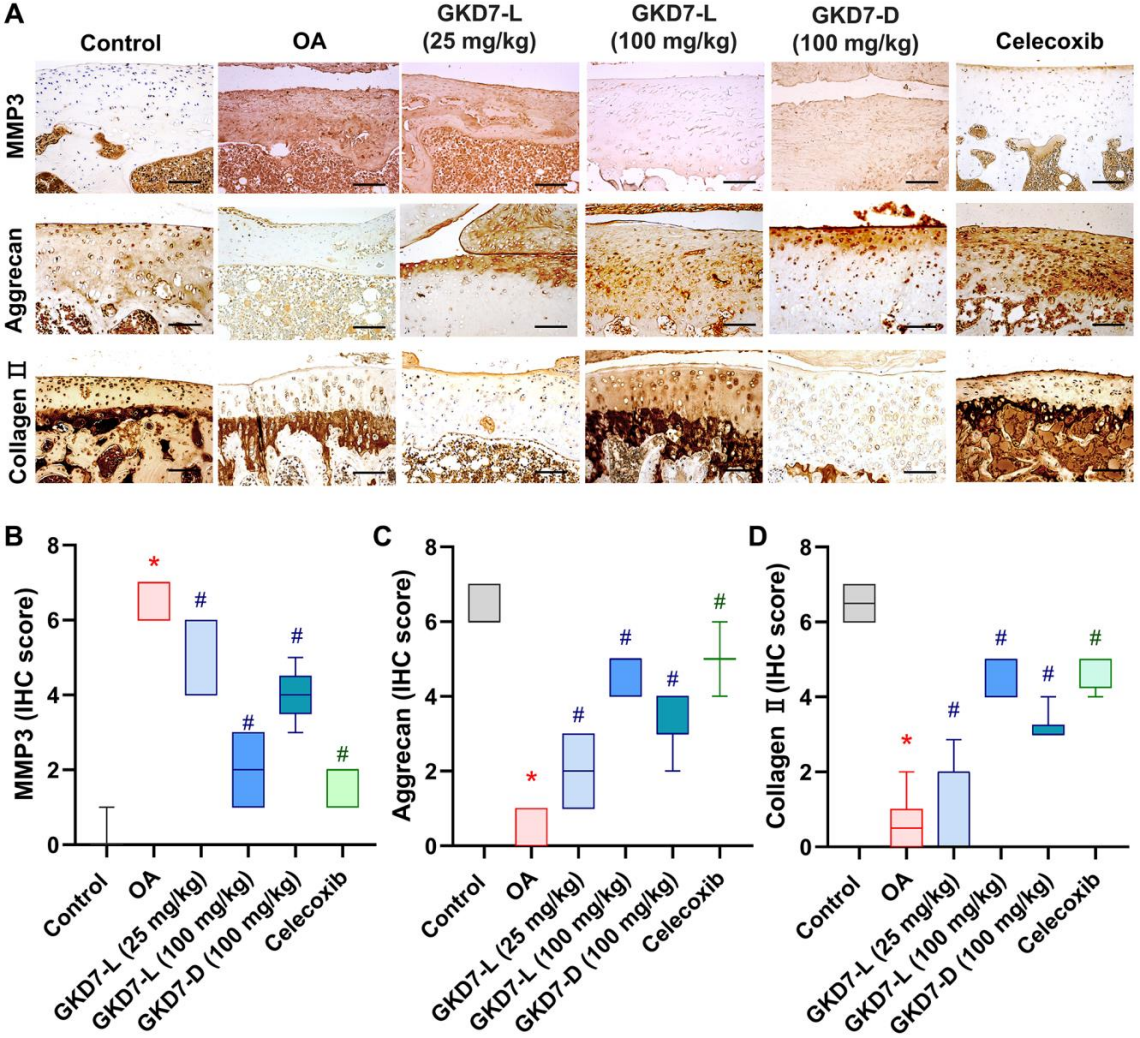
**Administration of GKD7-L and GKD7-D reduced levels of MMP3 and matrix degradation on cartilage.** (**A**) IHC staining of MMP3, aggrecan, and collagen II expression in a representative cartilage sample from the control, OA, OA+GKD7-L (25 mg/kg), OA+GKD7-L (100 mg/kg), and OA+GKD7-D (100 mg/kg) groups. Quantitative evaluations of cartilage’s MMP3, aggrecan, and collagen II (**B**–**D**). Values are presented as the mean ± SD for each group. Scale bar = 100 µm. ^*^p < 0.05 versus controls; ^#^*p* < 0.05 versus the OA group.

The previous studies demonstrated the IL-1β and TNF-α have important roles for mediating the synovial inflammation as well as the important role of MMP3 promoting other MMPs function to degrade the matrix of cartilage of OA [10–12, 15, 16]. Thus, we collected the knee tissue for detecting the expression of IL-1β and TNF-α on synovial tissue and MMP3 on cartilage by using immunohistochemical staining (Figure 5A). Our result shows that the increasing level of IL-1β and TNF-α was significantly reduced by oral administration of GKD7-L (25 or 100 mg/kg), GKD7-D (100 mg/kg) and celecoxib after ACLT surgery (Figure 5B, 5C). Additionally, our IHC data show that the joint’s cartilage has a reduction of IL-1β and TNF-α levels in GKD7 treatment groups (Supplementary Figures 1 and 2). The increasing level of MMP3 also significantly reduced by oral administration of GKD7-L (25 or 100 mg/kg), GKD7-D (100 mg/kg) and celecoxib (Figure 6A). And matrix markers, collage II and aggrecan which were decreasing by ACLT surgery also reversed by oral administration of GKD7-L (25 or 100 mg/kg), GKD7-D (100 mg/kg) and celecoxib (Figure 6B–6D). On the other hand, administration of GKD7 with different dosages and formulas decreased the collage II and aggrecan degradation through inhibiting the expression of MMP3, IL-1β and TNF-α on joint tissue. Additionally, our IHC data show that the joint’s synovial tissue has less MMP3 expression in GKD7 treatment groups (Supplementary Figure 3).

## DISCUSSION

OA is a chronic inflammatory disease which affects the pain behavior, synovial inflammation, and cartilage degradation [1, 2, 11, 12, 41, 42]. The proinflammatory cytokines, such as IL-1β and TNF-α, have important roles for mediating the progression of OA which cause joint pain, promote inflammatory response, and disturb chondrocyte metabolism of OA [11, 12, 41, 43]. Moreover, the clinical data which were reported in previous literatures indicated that there are significant higher levels of IL-1β and TNF-α which were expressed on synovial tissue and serum of OA patients [12, 27, 44]. In pre-clinical trial, IL-1β and TNF-α are also important targets to discover the useful therapeutic strategy for OA [12, 45, 46]. In present study, our data show that oral administration of GKD7-L (25 or 100 mg/kg), and GKD7-D (100 mg/kg) both effectively reduced IL-1β and TNF-α expression with reducing synovial inflammation. Although our previous study already demonstrated that administration of GKD7-L can slow down the progression of OA, in this study, GKD7-D also show the same effectivity with reducing synovial inflammation via IL-1β and TNF-α inhibition. Thus, GKD7 can apply to different applications for different dosage forms for OA treatment.

Chondroprotective effects are the main issue of the OA management. The matrix degradation of the cartilage is the major characteristic of OA, and MMP3 play the initiates which will be secreted by synovial cell and chondrocytes to promote MMPs degrading matrix of cartilage, such as collage II and aggrecan [47–49], synovial membrane hyperplasia, and promote osteoclast mature. Moreover, MMP3 also facilitate inflammatory cell accumulation, vascular invasion on bone and synovium and inhibit mesenchymal stem cell differentiation of OA [47]. Prior research has demonstrated that MMP3 is a member of the MMPs family, which is able to break down different cartilage matrix constituents. Furthermore, MMP3 has been identified as an important OA marker. According to mechanistic studies, MMP3 starts MMP-mediated degradation events in OA and acts as an activator for several other pro-MMPs. As a result, our main objective was to evaluate MMP3 expression in joint tissue [47]. The previous study also demonstrated that MMP3 inhibitor improves the progression of OA with reducing collage II degradation [50]. Moreover, the previous study also reported that MMP have been considered as important mark and function in synovial inflammation, which can degrade extracellular matrix with remodeling processes of arthritic disease [50]. Thus, MMP3 also have been considered as the important target in discovery for OA therapeutic strategy. In the present study, we also observe the high expression of MMP3 on ACLT-induced rat, and oral administration of GKD7-L and GKD7-D both significantly decrease the MMP3 expression with reversing the collage II and aggrecan degradation. Thus, GKD7 show the chondroprotective effects through inhibiting MMP3 activity with preventing collage II and aggrecan degradation in OA.

Numerous probiotic strains which were used as supplementations or health foods have shown therapeutic benefits of human illnesses, such as respiratory, gastrointestinal, and arthritis [51]. For treatment of OA, both preclinical and clinical studies strongly suggest that probiotics may benefit patients with OA pain through positive gut microbiota modulation and attenuating low-grade inflammation via multiple pathways [52]. And preclinical trial also suggests that probiotics inhibit cartilage damage and progression of OA model [53]. For example, administration of a mixture of probiotic strains *Lacticaseibacillus paracasei* 8700:2, *Lactiplantibacillus plantarum* HEAL9 and *L. plantarum* HEAL19 significantly inhibited cartilage damage at the medial femoral condyle on destabilization of medical meniscus mice model, and daily administration of *Streptococcus thermophilus* also improve the OA pain and cartilage damage on ACLT-induced rats model. And our previous research demonstrated that administration of GKD7-L suppresses ACLT-induced OA symptoms [25]. Live-type probiotics have been demonstrated in earlier research to improve gut microbiota, hence providing health advantages. Furthermore, live-type probiotics can constantly release advantageous compounds like postbiotics, which help to improve medical problems. Notably, earlier studies have shown that *L. plantarum* generates a large amount of polysaccharides, which have been shown to have anti-inflammatory effects on inflammation generated by LPS. These benefits of the live-type formula may allow the live-type GKD7 to have more effective OA treatment outcomes [25].

Previous studies have highlighted that IL-1β and TNF-α are major proinflammatory cytokines involved in OA. And these cytokines disturb chondrocyte metabolism by suppressing the synthesis of extracellular matrix proteins and stimulating the release of catabolic proteases. And MMP3 also have been reported as the as an activator for several other pro-MMPs and initiates MMP-mediated degradation reactions in OA. Thus, we propose that GKD7 can down-regulate MMP3 expression via reducing TNF-α, IL-1β expression on OA joint [47, 48]. For further investigation of the therapeutic effects of GKD7, our different dosage and dosage forms of GKD7 were all shown to significantly improve the OA pain and histopathological characteristic with inhibiting MMP3, IL-1β, and TNF-α expression to reverse collage II and aggrecan degradation in OA. These results suggest that GKD7-L and GKD7-D both as supplements can be used to improve OA progression and based on the same dosage (100 mg/kg) treatment, GKD7-L show more effective improving function. Higher dosage of GKD7 also show the similar therapeutic effects as the positive control celecoxib. Therefore, we suggest that GKD7 is a useful supplementation to serve as an alternative to celecoxib to avoid adverse effects.

In conclusion, our results show the oral administration of GKD7 with different dosage and dosage forms reducing the OA pain and OA joint destruction with improving weight-bearing posture and OA histopathological characteristics. And IHC staining result suggests that IL-1β and TNF-α expression on synovial tissue were reducing as well as the MMP3 expression reducing on cartilage, with prevention of the degradation of collage II and aggrecan by administration of GKD7-L and GKD7-D. Thus, we suggest that both GKD7-L and GKD7-D can be useful supplementation to OA improvement.

## Supplementary Materials

Supplementary Figures

## References

[1] Allen KD, Thoma LM, Golightly YM. Epidemiology of osteoarthritis. Osteoarthritis Cartilage. 2022; 30:184–95. 10.1016/j.joca.2021.04.020 34534661 PMC10735233

[2] Wieland HA, Michaelis M, Kirschbaum BJ, Rudolphi KA. Osteoarthritis - an untreatable disease? Nat Rev Drug Discov. 2005; 4:331–44. 10.1038/nrd1693 15803196

[3] Hunter DJ, Bierma-Zeinstra S. Osteoarthritis. Lancet. 2019; 393:1745–59. 10.1016/S0140-6736(19)30417-9 31034380

[4] Vina ER, Kwoh CK. Epidemiology of osteoarthritis: literature update. Curr Opin Rheumatol. 2018; 30:160–7. 10.1097/BOR.0000000000000479 29227353 PMC5832048

[5] Lin YY, Chen NF, Yang SN, Jean YH, Kuo HM, Chen PC, Feng CW, Liu YW, Lai YC, Wen ZH. Effects of *Streptococcus thermophilus* on anterior cruciate ligament transection-induced early osteoarthritis in rats. Exp Ther Med. 2021; 21:222. 10.3892/etm.2021.9653 33603831 PMC7851616

[6] de Liyis BG, Nolan J, Maharjana MA. Fibroblast growth factor receptor 1-bound extracellular vesicle as novel therapy for osteoarthritis. Biomedicine (Taipei). 2022; 12:1–9. 10.37796/2211-8039.1308 35836973 PMC9236721

[7] Goldring MB. Articular cartilage degradation in osteoarthritis. HSS J. 2012; 8:7–9. 10.1007/s11420-011-9250-z 23372517 PMC3295961

[8] Pap T, Korb-Pap A. Cartilage damage in osteoarthritis and rheumatoid arthritis--two unequal siblings. Nat Rev Rheumatol. 2015; 11:606–15. 10.1038/nrrheum.2015.95 26195338

[9] de Ruijter M, Diloksumpan P, Dokter I, Brommer H, Smit IH, Levato R, van Weeren PR, Castilho M, Malda J. Orthotopic equine study confirms the pivotal importance of structural reinforcement over the pre-culture of cartilage implants. Bioeng Transl Med. 2023; 9:e10614. 10.1002/btm2.10614 38193127 PMC10771555

[10] Liu S, Deng Z, Chen K, Jian S, Zhou F, Yang Y, Fu Z, Xie H, Xiong J, Zhu W. Cartilage tissue engineering: From proinflammatory and anti-inflammatory cytokines to osteoarthritis treatments (Review). Mol Med Rep. 2022; 25:99. 10.3892/mmr.2022.12615 35088882 PMC8809050

[11] Vincent TL. IL-1 in osteoarthritis: time for a critical review of the literature. F1000Res. 2019; 8:F1000. 10.12688/f1000research.18831.1 31249675 PMC6589928

[12] Su CH, Lin CY, Tsai CH, Lee HP, Lo LC, Huang WC, Wu YC, Hsieh CL, Tang CH. Betulin suppresses TNF-α and IL-1β production in osteoarthritis synovial fibroblasts by inhibiting the MEK/ERK/NF-κB pathway. J Funct Foods. 2021; 86:104729. 10.1016/j.jff.2021.104729

[13] Hou CH, Hsiao YC, Fong YC, Tang CH. Bone morphogenetic protein-2 enhances the motility of chondrosarcoma cells via activation of matrix metalloproteinase-13. Bone. 2009; 44:233–42. 10.1016/j.bone.2008.09.021 19038372

[14] Singh P, Marcu KB, Goldring MB, Otero M. Phenotypic instability of chondrocytes in osteoarthritis: on a path to hypertrophy. Ann N Y Acad Sci. 2019; 1442:17–34. 10.1111/nyas.13930 30008181

[15] Chakraborti S, Mandal M, Das S, Mandal A, Chakraborti T. Regulation of matrix metalloproteinases: an overview. Mol Cell Biochem. 2003; 253:269–85. 10.1023/a:1026028303196 14619979

[16] Echtermeyer F, Bertrand J, Dreier R, Meinecke I, Neugebauer K, Fuerst M, Lee YJ, Song YW, Herzog C, Theilmeier G, Pap T. Syndecan-4 regulates ADAMTS-5 activation and cartilage breakdown in osteoarthritis. Nat Med. 2009; 15:1072–6. 10.1038/nm.1998 19684582

[17] Mort JS, Billington CJ. Articular cartilage and changes in arthritis: matrix degradation. Arthritis Res. 2001; 3:337–41. 10.1186/ar325 11714387 PMC128908

[18] Sofat N. Analysing the role of endogenous matrix molecules in the development of osteoarthritis. Int J Exp Pathol. 2009; 90:463–79. 10.1111/j.1365-2613.2009.00676.x 19765101 PMC2768145

[19] Nagase H, Visse R, Murphy G. Structure and function of matrix metalloproteinases and TIMPs. Cardiovasc Res. 2006; 69:562–73. 10.1016/j.cardiores.2005.12.002 16405877

[20] Davey A, McAuley DF, O'Kane CM. Matrix metalloproteinases in acute lung injury: mediators of injury and drivers of repair. Eur Respir J. 2011; 38:959–70. 10.1183/09031936.00032111 21565917

[21] Lee CS, Kim SH. Anti-inflammatory and Anti-osteoporotic Potential of Lactobacillus plantarum A41 and L. fermentum SRK414 as Probiotics. Probiotics Antimicrob Proteins. 2020; 12:623–34. 10.1007/s12602-019-09577-y 31372901

[22] Sapra L, Dar HY, Bhardwaj A, Pandey A, Kumari S, Azam Z, Upmanyu V, Anwar A, Shukla P, Mishra PK, Saini C, Verma B, Srivastava RK. Lactobacillus rhamnosus attenuates bone loss and maintains bone health by skewing Treg-Th17 cell balance in Ovx mice. Sci Rep. 2021; 11:1807. 10.1038/s41598-020-80536-2 33469043 PMC7815799

[23] Martinez RM, Hulten KG, Bui U, Clarridge JE 3rd. Molecular analysis and clinical significance of Lactobacillus spp. recovered from clinical specimens presumptively associated with disease. J Clin Microbiol. 2014; 52:30–6. 10.1128/JCM.02072-13 24131686 PMC3911440

[24] Kullar R, Goldstein EJC, Johnson S, McFarland LV. *Lactobacillus* Bacteremia and Probiotics: A Review. Microorganisms. 2023; 11:896. 10.3390/microorganisms11040896 37110319 PMC10145752

[25] Lin YY, Chang SL, Liu SC, Achudhan D, Tsai YS, Lin SW, Chen YL, Chen CC, Chang JW, Fong YC, Hu SL, Tang CH. Therapeutic Effects of Live *Lactobacillus plantarum* GKD7 in a Rat Model of Knee Osteoarthritis. Nutrients. 2022; 14:3170. 10.3390/nu14153170 35956346 PMC9370768

[26] Chen H, Ma X, Liu Y, Ma L, Chen Z, Lin X, Si L, Ma X, Chen X. Gut Microbiota Interventions With *Clostridium butyricum* and Norfloxacin Modulate Immune Response in Experimental Autoimmune Encephalomyelitis Mice. Front Immunol. 2019; 10:1662. 10.3389/fimmu.2019.01662 31428083 PMC6689973

[27] Ko CY, Lin YY, Achudhan D, Chang JW, Liu SC, Lai CY, Huang YL, Tsai CH, Fong YC, Chen HT, Lee KT, Huang CC, Chang TK, Tang CH. Omentin-1 ameliorates the progress of osteoarthritis by promoting IL-4-dependent anti-inflammatory responses and M2 macrophage polarization. Int J Biol Sci. 2023; 19:5275–89. 10.7150/ijbs.86701 37928270 PMC10620827

[28] Lee HP, Liu SC, Wang YH, Chen BC, Chen HT, Li TM, Huang WC, Hsu CJ, Wu YC, Tang CH. Cordycerebroside A suppresses VCAM-dependent monocyte adhesion in osteoarthritis synovial fibroblasts by inhibiting MEK/ERK/AP-1 signaling. J Funct Foods. 2021; 86:104712. 10.1016/j.jff.2021.104712

[29] Wang YH, Kuo SJ, Liu SC, Wang SW, Tsai CH, Fong YC, Tang CH. Apelin Affects the Progression of Osteoarthritis by Regulating VEGF-Dependent Angiogenesis and miR-150-5p Expression in Human Synovial Fibroblasts. Cells. 2020; 9:594. 10.3390/cells9030594 32131466 PMC7140420

[30] Hou CH, Lin FL, Hou SM, Liu JF. Cyr61 promotes epithelial-mesenchymal transition and tumor metastasis of osteosarcoma by Raf-1/MEK/ERK/Elk-1/TWIST-1 signaling pathway. Mol Cancer. 2014; 13:236. 10.1186/1476-4598-13-236 25326651 PMC4210521

[31] Chang SL, Lin YY, Liu SC, Tsai YS, Lin SW, Chen YL, Chen CC, Ko CY, Chen HT, Chen WC, Tang CH. Oral Administration of *Clostridium butyricum* GKB7 Ameliorates Signs of Osteoarthritis in Rats. Cells. 2022; 11:2169. 10.3390/cells11142169 35883610 PMC9323988

[32] Lee KT, Su CH, Liu SC, Chen BC, Chang JW, Tsai CH, Huang WC, Hsu CJ, Chen WC, Wu YC, Tang CH. Cordycerebroside A inhibits ICAM-1-dependent M1 monocyte adhesion to osteoarthritis synovial fibroblasts. J Food Biochem. 2022; 46:e14108. 10.1111/jfbc.14108 35165902

[33] Achudhan D, Li-Yun Chang S, Liu SC, Lin YY, Huang WC, Wu YC, Huang CC, Tsai CH, Ko CY, Kuo YH, Tang CH. Antcin K inhibits VCAM-1-dependent monocyte adhesion in human rheumatoid arthritis synovial fibroblasts. Food Nutr Res. 2022; 66. 10.29219/fnr.v66.8645 35783555 PMC9210827

[34] Lee HP, Wu YC, Chen BC, Liu SC, Li TM, Huang WC, Hsu CJ, Tang CH. Soya-cerebroside reduces interleukin production in human rheumatoid arthritis synovial fibroblasts by inhibiting the ERK, NF-κB and AP-1 signalling pathways. Food and Agricultural Immunology. 2020; 31:740–50. 10.1080/09540105.2020.1766426

[35] Hou CH, Yang RS, Tsao YT. Connective tissue growth factor stimulates osteosarcoma cell migration and induces osteosarcoma metastasis by upregulating VCAM-1 expression. Biochem Pharmacol. 2018; 155:71–81. 10.1016/j.bcp.2018.06.015 29909077

[36] Liu JF, Chen PC, Chang TM, Hou CH. Monocyte Chemoattractant Protein-1 promotes cancer cell migration via c-Raf/MAPK/AP-1 pathway and MMP-9 production in osteosarcoma. J Exp Clin Cancer Res. 2020; 39:254. 10.1186/s13046-020-01756-y 33228783 PMC7684958

[37] Zhang J, Fu B, Chen X, Chen D, Yang H. Protocatechuic acid attenuates anterior cruciate ligament transection-induced osteoarthritis by suppressing osteoclastogenesis. Exp Ther Med. 2020; 19:232–40. 10.3892/etm.2019.8189 31853294 PMC6909799

[38] Oláh T, Cai X, Gao L, Walter F, Pape D, Cucchiarini M, Madry H. Quantifying the Human Subchondral Trabecular Bone Microstructure in Osteoarthritis with Clinical CT. Adv Sci (Weinh). 2022; 9:e2201692. 10.1002/advs.202201692 35670136 PMC9376842

[39] Mambrini M, Mecozzi L, Ferrini E, Leo L, Bernardi D, Grandi A, Sverzellati N, Ruffini L, Silva M, Stellari FF. The importance of routine quality control for reproducible pulmonary measurements by in vivo micro-CT. Sci Rep. 2022; 12:9695. 10.1038/s41598-022-13477-7 35690601 PMC9188608

[40] Sanchez-Lopez E, Coras R, Torres A, Lane NE, Guma M. Synovial inflammation in osteoarthritis progression. Nat Rev Rheumatol. 2022; 18:258–75. 10.1038/s41584-022-00749-9 35165404 PMC9050956

[41] Li H, Xie S, Qi Y, Li H, Zhang R, Lian Y. TNF-α increases the expression of inflammatory factors in synovial fibroblasts by inhibiting the PI3K/AKT pathway in a rat model of monosodium iodoacetate-induced osteoarthritis. Exp Ther Med. 2018; 16:4737–44. 10.3892/etm.2018.6770 30542428 PMC6257214

[42] Hou CH, Fong YC, Tang CH. HMGB-1 induces IL-6 production in human synovial fibroblasts through c-Src, Akt and NF-κB pathways. J Cell Physiol. 2011; 226:2006–15. 10.1002/jcp.22541 21520052

[43] Hou CH, Tang CH, Hsu CJ, Hou SM, Liu JF. CCN4 induces IL-6 production through αvβ5 receptor, PI3K, Akt, and NF-κB singling pathway in human synovial fibroblasts. Arthritis Res Ther. 2013; 15:R19. 10.1186/ar4151 23343403 PMC3672729

[44] Lee KT, Chen BC, Liu SC, Lin YY, Tsai CH, Ko CY, Tang CH, Tung KC. Nesfatin-1 facilitates IL-1β production in osteoarthritis synovial fibroblasts by suppressing miR-204-5p synthesis through the AP-1 and NF-κB pathways. Aging (Albany NY). 2021; 13:22490–501. 10.18632/aging.203559 34560673 PMC8507299

[45] Gungor H, Ekici M, Onder Karayigit M, Turgut NH, Kara H, Arslanbas E. Zingerone ameliorates oxidative stress and inflammation in bleomycin-induced pulmonary fibrosis: modulation of the expression of TGF-β1 and iNOS. Naunyn Schmiedebergs Arch Pharmacol. 2020; 393:1659–70. 10.1007/s00210-020-01881-7 32377772

[46] Ren C, Jin J, Hu W, Chen Q, Yang J, Wu Y, Zhou Y, Sun L, Gao W, Zhang X, Tian N. Betulin Alleviates the Inflammatory Response in Mouse Chondrocytes and Ameliorates Osteoarthritis via AKT/Nrf2/HO-1/NF-κB Axis. Front Pharmacol. 2021; 12:754038. 10.3389/fphar.2021.754038 34721040 PMC8548689

[47] Wan J, Zhang G, Li X, Qiu X, Ouyang J, Dai J, Min S. Matrix Metalloproteinase 3: A Promoting and Destabilizing Factor in the Pathogenesis of Disease and Cell Differentiation. Front Physiol. 2021; 12:663978. 10.3389/fphys.2021.663978 34276395 PMC8283010

[48] Kiani C, Chen L, Wu YJ, Yee AJ, Yang BB. Structure and function of aggrecan. Cell Res. 2002; 12:19–32. 10.1038/sj.cr.7290106 11942407

[49] Hou CH, Chiang YC, Fong YC, Tang CH. WISP-1 increases MMP-2 expression and cell motility in human chondrosarcoma cells. Biochem Pharmacol. 2011; 81:1286–95. 10.1016/j.bcp.2011.03.016 21453685

[50] Ni GX, Zhan LQ, Gao MQ, Lei L, Zhou YZ, Pan YX. Matrix metalloproteinase-3 inhibitor retards treadmill running-induced cartilage degradation in rats. Arthritis Res Ther. 2011; 13:R192. 10.1186/ar3521 22114772 PMC3334642

[51] West NP, Horn PL, Pyne DB, Gebski VJ, Lahtinen SJ, Fricker PA, Cripps AW. Probiotic supplementation for respiratory and gastrointestinal illness symptoms in healthy physically active individuals. Clin Nutr. 2014; 33:581–7. 10.1016/j.clnu.2013.10.002 24268677

[52] Rahman SO, Bariguian F, Mobasheri A. The Potential Role of Probiotics in the Management of Osteoarthritis Pain: Current Status and Future Prospects. Curr Rheumatol Rep. 2023; 25:307–26. 10.1007/s11926-023-01108-7 37656392 PMC10754743

[53] Sophocleous A, Azfer A, Huesa C, Stylianou E, Ralston SH. Probiotics Inhibit Cartilage Damage and Progression of Osteoarthritis in Mice. Calcif Tissue Int. 2023; 112:66–73. 10.1007/s00223-022-01030-7 36261653 PMC9813193

