# Supplementary material for: The joint protective function of live- and dead-Lactobacillus plantarum GKD7 on anterior cruciate ligament transection induces osteoarthritis
Source: Aging (Albany NY). 2024 Sep 5;16(18):12559–73. doi: 10.18632/aging.206101 (PMC11466490; doi:10.18632/aging.206101)
Supplement: Supplementary Figures [file aging-16-206101-s001.pdf]

SUPPLEMENTARY FIGURES

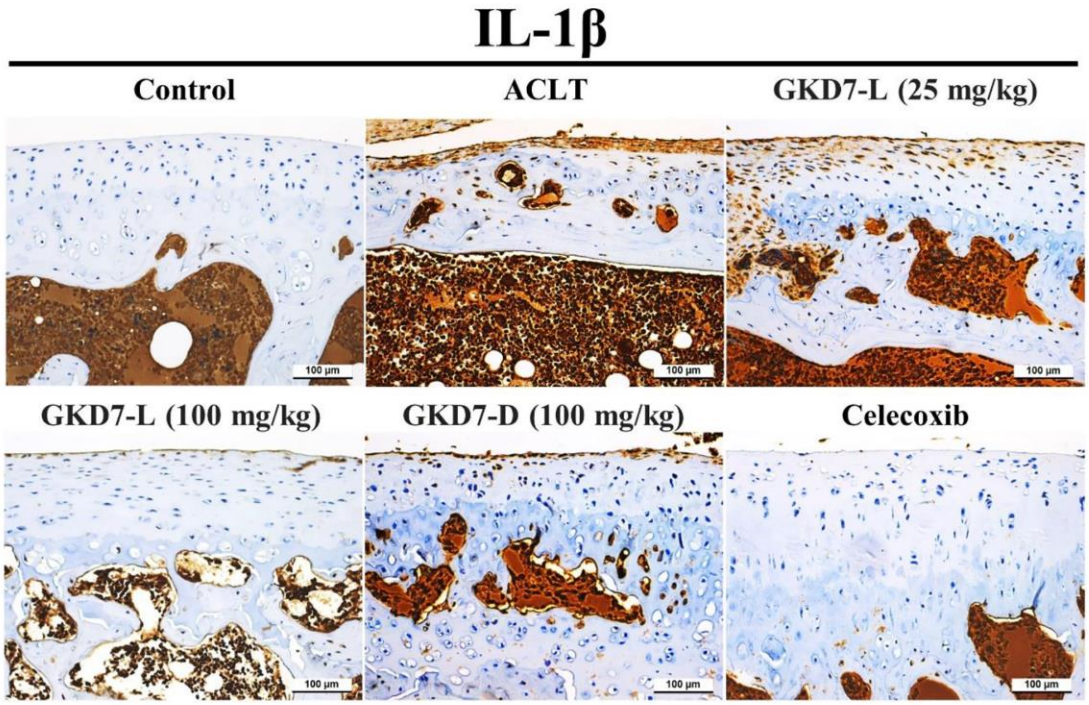

Supplementary Figure 1. GKD7-L and GKD7-D reduce ACLT-induced IL-1β production in cartilage. Histological sections of joints were IHC stained with IL-1β. Scale bar = 100 µm.

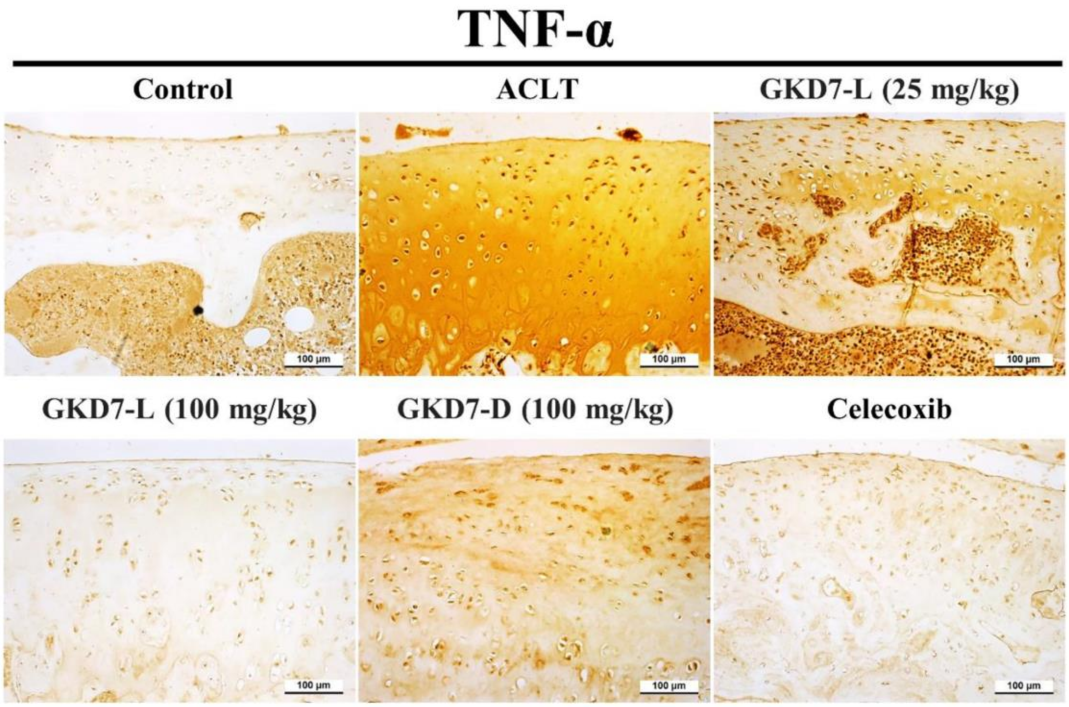

Supplementary Figure 2. GKD7-L and GKD7-D reduce ACLT-induced TNF-α production in cartilage. Histological sections of joints were IHC stained with TNF-α.

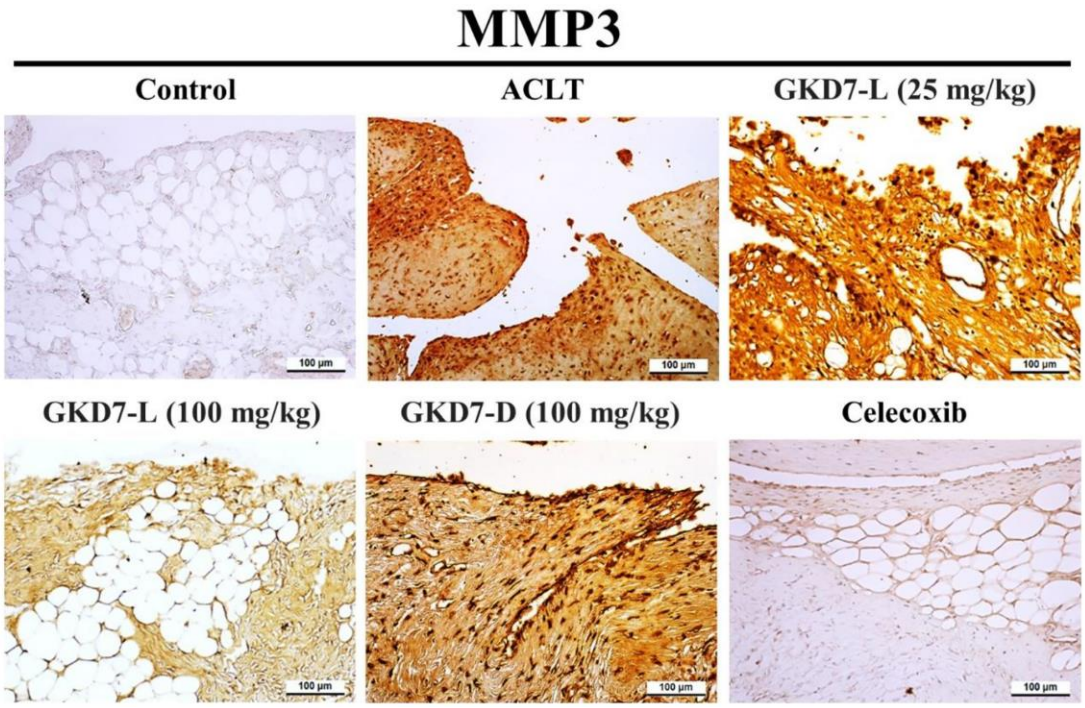

Supplementary Figure 3. GKD7-L and GKD7-D reduce ACLT-induced MMP3 production in synovial tissue. Histological sections of joints were IHC stained with MMP3. Scale bar = 100 μm.
